# Development of a surrogate potency assay to determine the angiogenic activity of Stempeucel®, a pooled, ex-vivo expanded, allogeneic human bone marrow mesenchymal stromal cell product

**DOI:** 10.1186/s13287-017-0488-3

**Published:** 2017-02-28

**Authors:** Charan Thej, Balamurugan Ramadasse, Ankita Walvekar, Anish S. Majumdar, Sudha Balasubramanian

**Affiliations:** 1 Stempeutics Research Pvt. Ltd, Akshay Tech Park, #72 & 73, 2nd Floor, EPIP Zone, Phase 1, Bangalore, Whitefield 560066 India; 2 Manipal University, Manipal, Karnataka India

**Keywords:** Potency assay, Angiogenesis, Pooled human bone marrow-derived mesenchymal stromal cell, Vascular endothelial growth factor, Endothelial tube formation

## Abstract

**Background:**

Mesenchymal stromal cells (MSCs) have emerged as a more beneficial alternative to conventional therapy and may offer a potential cure for unmet medical needs. MSCs are known to possess strong immunomodulatory and anti-inflammatory properties. Moreover, they promote angiogenesis and tissue regeneration through the secretion of trophic factors. For these reasons, the past decade witnessed a sharp increase in the number of clinical trials conducted with stem cells for various vascular diseases requiring angiogenesis. In this study, we evaluated the in vitro angiogenic potency of Stempeucel®, which is an allogeneic pooled human bone marrow-derived mesenchymal stromal cell (phBMMSC) product. We previously established the safety of Stempeucel® in our pre-clinical studies, and clinical trials conducted for critical limb ischaemia and acute myocardial infarction.

**Methods:**

Because the proposed mechanism of action of phBMMSCs is mainly through the secretion of pro-angiogenic cytokines, we developed a surrogate potency assay by screening various batches of large-scale expanded phBMMSCs for the expression of angiogenic factors and cytokines through gene expression and growth factor analyses, followed by in vitro functional assays.

**Results:**

The well characterized angiogenic vascular endothelial growth factor (VEGF) was selected and quantified in twenty six manufactured batches of phBMMSCs to establish consistency following the United States Food and Drug Administration recommendations. According to recommendations 21 CFR 211.165(e) and 211.194(a)(2), we also established and documented the specificity and reproducibility of the test methods employed through validation. Moreover, we also attempted to elucidate the mechanism of action of the cell population to ensure appropriate biological activity. The functional role of VEGF has been established through in vitro angiogenic assays and a dose-dependent correlation was observed with in vitro functional results.

**Conclusions:**

The data generated from this study suggest the selection of VEGF as a single surrogate marker to test the angiogenic potency of phBMMSCs. Our study reports the quantification of VEGF in twenty six batches of large-scale manufactured phBMMSCs, and a concentration-dependent correlation of secreted VEGF to endothelial cell functions of migration, proliferation and tube formation, in the conditioned medium obtained from nine phBMMSC batches. To our cognizance, this is the first study in which a single angiogenic factor (VEGF) has been qualified as a surrogate potency marker through all three in vitro functional assays to determine the angiogenic potency of the phBMMSC population.

**Electronic supplementary material:**

The online version of this article (doi:10.1186/s13287-017-0488-3) contains supplementary material, which is available to authorized users.

## Background

Angiogenesis is a physiological process of new blood vessel formation from pre-existing vasculature, playing an orchestrated role in the growth and development of tissues and organs as well as in the process of wound healing [1]. In tissues requiring revascularization, endothelial cells migrate, proliferate and differentiate to form primitive tubular networks, which are regulated by pro-angiogenic growth factors and cytokines [2]. The primitive tubular network requires maturation and stability, which is controlled by a ‘dynamic angiogenic balance’, regulated by angiogenic stimulators and inhibitors [3]. The induction of new vasculature is stimulated either by the administration of pro-angiogenic factors through recombinant proteins/genes or by progenitor cell populations, which could secrete multiple pro-angiogenic cytokines [4]. Both of these approaches have been well tolerated, although larger clinical trial data are required to demonstrate the safety and efficacy of such therapy using optimal delivery methods [5]. Varying degrees of success in cell-based therapeutic angiogenesis have been achieved by the intramuscular administration of endothelial progenitor cells (EPCs) [6, 7], peripheral blood mononuclear cells (PBMNCs) [8], bone marrow mononuclear cells (BMMNCs) [9–11] and bone marrow-derived mesenchymal stromal cells (BMMSCs) [12].

Recently, mesenchymal stromal cells (MSCs) obtained from various tissues have gained importance in regenerative medicine because of their multipotent activity, including short-term engraftment, paracrine function and differentiation to endothelial cells [13–15]. Among various tissues, BMMSCs have been extensively characterized and used in clinical studies due to their vast ex-vivo expansion potential and their ability to perform multiple biological functions simultaneously to promote tissue regeneration in vivo [16]. In this study, we progressively developed an angiogenic potency assay for Stempeucel®, which consists of adult human bone marrow-derived, pooled, culture-expanded MSCs (pooled human BMMSCs (phBMMSCs)). The development and characterization of the phBMMSC population has been published previously [17–20]. Owing to the fact that striking variations have been observed in the secretome profile of different donors, we have developed a pooling technology to minimize such donor-specific variability and have generated data demonstrating that a pooled MSC population is more consistent and stable in secreting angiogenic and other cytokines that are critical for regenerative capacity of these cells in comparison with single donor-derived MSCs (Thej et al., manuscript in preparation). In addition, our published clinical trials conducted in critical limb ischaemia (CLI) patients using phBMMSCs clearly demonstrated that these pooled cells were safe [19] and therapeutically effective in patients suffering from CLI due to Buerger’s disease [20].

As a global industrial standard for cell therapy products (CTPs), the development of in vitro potency assays based on the mechanism of action for a particular disease indication in large-scale expanded cells becomes essential. Potency is one of the critical quality control measures required for a CTP to demonstrate its biological activity, which should ideally be related to the intended therapeutic response. The probable mechanism of action by which MSCs elicit an angiogenic response is through their paracrine activity [14, 21]. The secretion of pro-angiogenic and vascular stability factors, such as vascular endothelial growth factor (VEGF), angiopoietin-1 (Ang-1), stromal-derived factor-1 (SDF-1), interleukin (IL)-8, IL-6, hepatocyte growth factor (HGF) and transforming growth factor beta-1 (TGF-β1), play a role in the recruitment of endogenous endothelial cells which subsequently undergo proliferation and differentiation into new blood vessels both in vitro and in vivo [22, 23]. In order to develop a suitable angiogenic potency assay for Stempeucel® based on the reported mechanism of action, we screened the cells for the expression of widely reported pro-angiogenic genes and further quantified the corresponding growth factors in various batches of large-scale expanded allogeneic phBMMSCs. In due course we selected VEGF – a single, consistently expressed angiogenic factor and one of the most extensively studied growth factors responsible for angiogenesis – as the surrogate potency marker, and further validated its mechanism of action and in vitro functional role.

## Methods

### Cell culture

Stempeucel® is a bone marrow-derived, ex-vivo expanded, pooled, allogeneic human MSC population that has been characterized previously [17, 24] (Additional file 1: Figure S1). Briefly, BMMNCs were separated from the bone marrow by the Ficoll density gradient method (1.077 g/ml density). BMMNCs accumulated at the buffy coat layer were plated into T-75 flasks (BD Biosciences, San Jose, CA, USA) in the medium comprising DMEM-KO (Gibco, Carlsbad, CA, USA), 10% fetal bovine serum (FBS; Hyclone, Waltham, MA, USA), 100 U/ml of Penstrep (Gibco) and 100 U/ml of Glutamax (Gibco). BMMNCs from three different donors were expanded individually up to passage 1 (P1). The cells from P1 were pooled in an equal ratio and plated for expansion to subsequent passages at a plating density of 1000 cells/cm^2^. The addition of 2 ng/ml of basic fibroblast growth factor (bFGF; Sigma-Aldrich, St. Louis, MO, USA) was introduced into the medium at P2. The cells were further sub-cultured up to five passages in 10 cell stacks (Corning, NY, USA). The pooled allogeneic human BMMSC population at P5 was considered Stempeucel®.

Human foreskin fibroblasts (HFFs; Lonza, Switzerland) were cultured in DMEM-KO, 10% FBS, 100 U/ml of Penstrep and 100 U/ml of Glutamax. HFFs were used for assays at P6 and P7.

Umbilical cords for isolating human umbilical vein endothelial cells (HUVECs) were obtained with the informed consent of the donors using the guidelines approved by the Institutional Committee for Stem Cell Research and Therapy and Institutional Ethics Committee at Manipal Hospital, Bangalore, India. The umbilical vein was washed three times with HBSS, and endothelial cells from the basement membrane were detached using 0.2% collagenase 1 (Sigma-Aldrich). Cells were cultured in endothelial growth medium 2 (EGM-2; Lonza), using 0.2% gelatin (Sigma-Aldrich) as an attachment substrate. The cells were characterized for endothelial markers, CD31, CD34, VE-Cadherin and Von Willebrand factor (vWF) by flow cytometry (BD LSR II; BD Biosciences) and gene expression by reverse transcriptase PCR (Additional file 2: Figure S2). All cells were grown at 37 °C in a 5% CO_2_ incubator (Binder, Bohemia, NY, USA).

### Real-time polymerase chain reaction

Total cellular RNA was isolated using an RNeasy mini kit (Qiagen, Hilden, Germany). The RNA samples were treated with RNAase-free *DNase I* (Ambion, Thermo Fischer Scientific, Waltham, MA, USA) according to the manufacturer’s instructions and were reverse-transcribed into cDNA using a high-capacity cDNA reverse transcription kit (Applied Biosystems, Foster City, CA, USA) according to the manufacturer’s instructions. The transcripts were amplified using SYBR green (Applied Biosystems). The gene-specific primers and primer sequences are presented in Table 1. A reverse transcriptase negative control was taken from each sample, and a no-template blank served as the negative control. Gene expression was normalized to the housekeeping gene β-Actin which was used as an internal standard. Subsequently, ^ΔΔ^CT was calculated against a fibroblast cell line, HFF. Similarly, VEGFR1, VEGFR2 and VEGFR3 expression in HUVECs was normalized against the same cells grown in endothelial growth medium (EGM) and expressed as the fold-change.

**Table 1:** Primer list- Real Time PCR

Markers	Forward primer	Reverse primer	Tm	Product length
β-Actin	CATGTACGTTGCTATCCAGGC	CTCCTTAATGTCACGCACGAT	60	250bp
VEGF-A	GCAGAATCATCACGAAGTGG	GCATGGTGATGTTGGACTCC	60	234bp
TGF-β1	CAGATCCTGTCCAAGCTG	TCGGAGCTCTGATGGTGTT	56	270bp
Ang-1	GCTTACCAGATTCACACTGTTCC	TTGCTACCTTGCCAACAACAACTG	60	612bp
IL-6	CACAGACAGCCACTCACCTC	TTTTCTGCCAGTGCCTCTTT	60	137bp
HGF	ATGCATCCAAGGTCAAGGAG	TTCCATGTTCTTGTCCCACA	61	349bp
SDF-1α/CXCL12	TGCCAGAGCCAACGTCAAG	CAGCCGGGCTACAATCTGAA	60	73bp
VEGFR1	CGTAGAGATGTACAGTGAAA	GGTGTGCTTATTTGGACATC	55	306bp
VEGFR2	TTACAGATCTCCATTTATTGC	TTCATCTCACTCCCAGACT	60	498bp
VEGFR3	CTGGACCGAGTTTGTGGAGG	GTCACATAGAAGTAGATGAGCCG	60	138bp

*Tm* melting temperature, *VEGF-A* vascular endothelial growth factor A, *TGF-β* transforming growth factor beta, *Ang-1* angiopoietin-1, *IL* interleukin, *HGF* hepatocyte growth factor, *SDF-1* stromal-derived factor-1, *VEGFR1* vascular endothelial growth factor receptor 1, *VEGFR2* vascular endothelial growth factor receptor 2, *VEGFR3* vascular endothelial growth factor receptor 3

### Preparation and collection of the conditioned medium from phBMMSCs

phBMMSCs from twenty six different batches were thawed and plated at a density of 1 × 10^6^ cells per T-75 flask in duplicates. The cultures were fed with 10 ml of DMEM-KO, 10% FBS, 100 U/ml of Glutamax, 100 U/ml of Penstrep and 2 ng/ml of bFGF. The conditioned medium (CM) was collected at the end of 48 and 72 h from the T-75 flasks and used for growth factor estimations and in vitro functional assays. Complete medium without cells was also incubated for 72 h and used as a control for background determination. The collected media were spun down at 1500 rpm for 5 min to eliminate cell debris, filtered through a 0.22-μm syringe filter (Merck-Millipore, NJ, USA), aliquoted and stored at –80 °C until they were used for subsequent assays.

### Secretome analysis by growth factor array

The control medium and CM from six phBMMSC batches were analysed for the presence of cytokines, chemokines and growth factors by performing a semi-quantitative human growth factor antibody-based array (RayBio® Human Growth Factor Array AAH-GF-1-8; Ray Biotech, Norcross GA, USA). The experiment was performed as per the manufacturer’s instructions and chemiluminescence was recorded using ImageQuant LAS 4000 (GE Healthcare, MA, USA). The data recorded were analysed using ImageJ software (NIH). The relative intensities of individual growth factors were calculated as arbitrary units after background correction and normalized to blot intensities obtained with the control medium. The complete array layout is depicted in Additional file 3: Table S1a.

### Enzyme-linked immunosorbent assay

Human angiogenic cytokines, VEGF, Ang-1, SDF-1α, IL-6, IL-8, HGF and TGF-β1 in the CM were estimated using enzyme-linked immunosorbent assay (ELISA) Kits (R&D Systems, Minneapolis, MN, USA) according to the manufacturer’s directions. Control medium was also assayed in each ELISA; any non-specific detection of growth factors/cytokines was subtracted from the respective CM values. The samples were assayed in duplicates.

### Cell migration assay

The cell migration assay was performed using a Transwell two-chamber cell culture method and Transwell inserts (Corning, Cambridge, MA, USA) with an 8-μm-pore polycarbonate membrane. HUVECs were serum starved for 18 h in the endothelial basal medium (EBM) supplemented with 0.1% FBS. The cells were then trypsinized and reconstituted in the EBM at a density of 1 × 10^5^ cells/100 μl. Next, 1 × 10^5^ cells/well were plated into the upper chamber of the Transwell insert. In the lower chamber, 0.65 ml of EGM, complete medium, serum-free medium (SFM) or CM from phBMMSC was added. The phBMMSC CM with 10 μg/ml of monoclonal mouse IgG anti-human VEGF neutralizing antibody (Clone # 26503; R&D Systems) or IgG isotype control (Abcam, Cambridge, MA, USA) was used for additional conditions. HUVECs were allowed to migrate for 18 h at 37 °C in a humidified incubator. The non-migrated cells were removed carefully using a pre-wet cotton swab, following which the membrane was fixed with 4% paraformaldehyde and stained with haematoxylin for 10 min. After washing, the membrane was mounted on a slide with Dibutylphthalate Polystyrene Xylene (DPX; Merck-Millipore, MA, USA), and the cells that migrated from the upper to the lower side of the membrane were counted under a bright-field microscope at 100× objective (100× magnification, Nikon 90*i* microscope). All of the assays were performed in triplicates.

### Cell proliferation assay

HUVECs were plated at a density of 2 × 10^3^ cells/well in 96-well plates and cultured for 24 h in 200 μl/well of the EGM. The cells were then washed twice with HBSS and serum-starved for 18 h in the EBM supplemented with 0.1% FBS. The serum starvation medium was replaced with equal volumes of EGM, control medium, CM, CM containing anti-VEGF neutralizing antibody (10 μg/ml) or IgG isotype control (10 μg/ml), SFM or Axitinib (0.25 nM, 0.29 nM and 1.2 nM) (Cayman Chemical, MI, USA). HUVECs were allowed to proliferate for 72 h. Then 5-bromo-2′-deoxyuridine (100 μM) was added (1:1000 in SFM) at the 48th hour, and its uptake by the cells was measured using a colorimetric cell proliferation ELISA kit (Merck-Millipore, NJ, USA).

To determine the effect of CM on VEGFR1, VEGFR2 and VEGFR3 gene expression, HUVECs were plated at a density of 5 × 10^3^ cells/cm^2^ in six-well dishes (BD Bioscience) in EGM. The following day, HUVECs were serum starved for 18 h in EBM supplemented with 0.1% FBS. CM from two batches of phBMMSCs were incubated alone or with anti-VEGF antibody (10 μg/ml) on HUVECs, and with EGM, which served as control. HUVECs were allowed to proliferate for 72 h. The cells were then harvested and processed further for RT-PCR analysis.

### In vitro endothelial tube formation assay

Growth factor-reduced (GFR) Matrigel (BD Bioscience) was thawed on ice at 4 °C overnight. Ten microlitres of Matrigel was plated onto each inner well of the μ-angiogenesis slides (IBIDI, Germany) and allowed to solidify at 37 °C for 30 min. 1 × 10^4^ HUVECs were reconstituted in CM only, CM containing anti-VEGF neutralizing antibody (10 μg/ml), CM with IgG isotype control (10 μg/ml)﻿, complete medium, EGM or SFM to a total volume of 50 μl and plated on the GFR Matrigel. The plates were incubated for 6 h in a humidified incubator at 5% CO_2_ and 37 °C. Images were taken using an inverted phase-contrast microscope (Nikon) under 4× and 10× objectives. The tube length was measured using WimTube (Wimasis, GmbH, Germany) from the 4× magnification images of three wells for each condition. The experimental samples and controls were assayed in triplicates.

### Statistical analysis

The data were analysed using GraphPad Prism 5 software (GraphPad Software, Inc., La Jolla, CA, USA; http://www.graphpad.com). Pairs of data sets were analysed for statistical significance using one-way ANOVA, linear regression analysis or Student’s *t* test (95% confidence interval). *p* < 0.05 was considered significant. The results were presented as means ± SEM.

## Results

### Screening the angiogenic predisposition of phBMMSCs

**Fig. 1:**
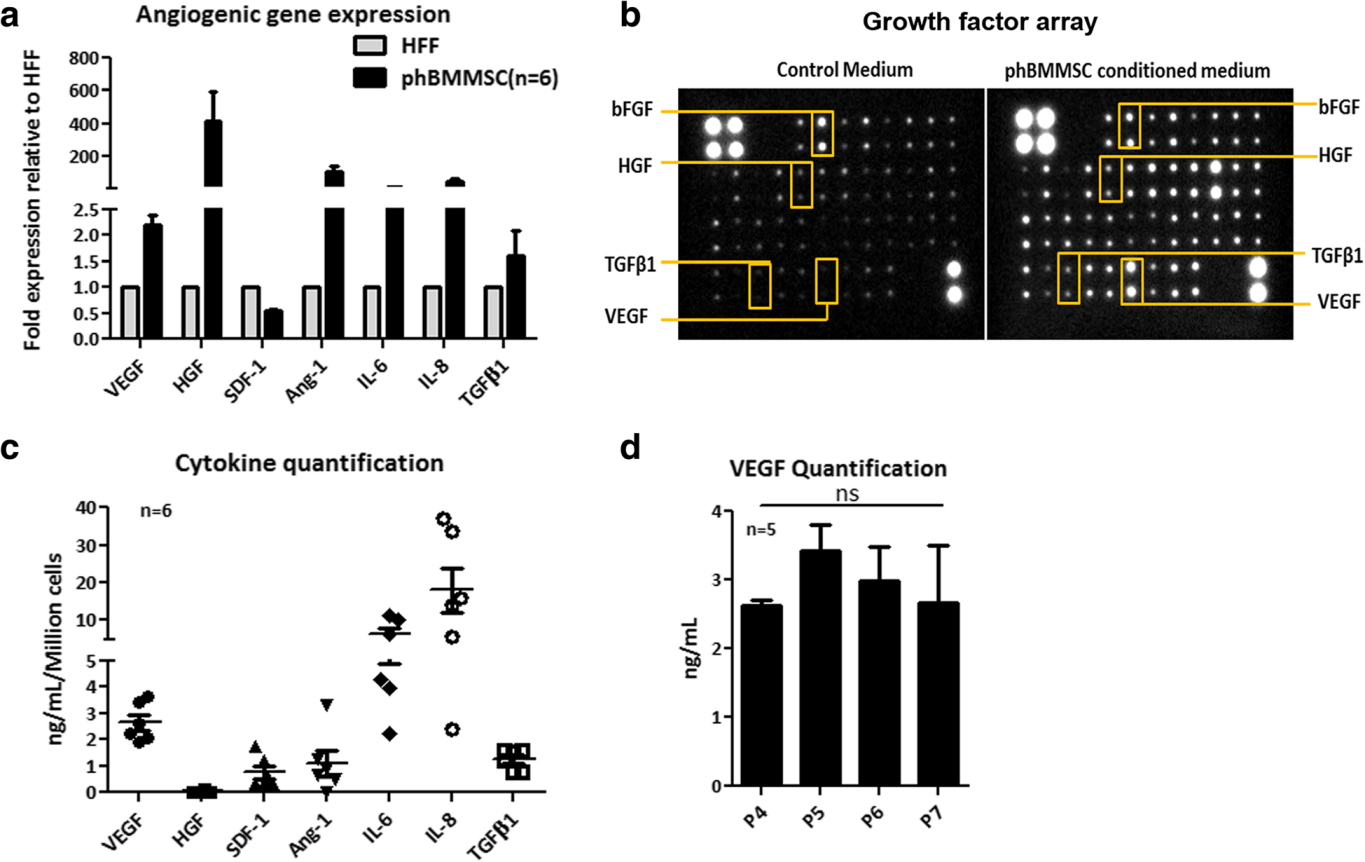
**a** qRT-PCR analysis of seven angiogenic genes: VEGF, HGF, SDF-1, Ang-1, IL-6, IL-8 and TGF-β1 in six batches of phBMMSCs. Relative fold expression was depicted after normalization to the housekeeping gene β-Actin as an internal standard and subsequent normalization using human foreskin fibroblast (*HFF*) cells for the same genes. **b** Secretome analysis of control medium and phBMMSC CM using 41 growth factor antibody arrays detected by chemiluminescence; a few detected growth factors marked in *boxes*. **c** Quantification of seven selected angiogenic growth factors/cytokines using human-specific ELISAs in same six batches of phBMMSCs. **d** Quantification of VEGF by ELISA in CM from five batches of phBMMSCs from P4 to P7; one-way ANOVA revealed no significant difference in VEGF levels between P4 and P7, *p* = 0.699. Data presented as mean ± SEM. SEMs shown as *error bars. phBMMSC* pooled human bone marrow derived mesenchymal stromal cell, *Ang-1* angiopoietin-1, *SDF-1* stromal-derived factor-1, *bFGF* basic fibroblast growth factor, *HGF* hepatocyte growth factor, *IL* interleukin, *TGFβ1* transforming growth factor beta-1, *VEGF* vascular endothelial growth factor

Human BMMSCs are known to express and secrete a battery of pro-angiogenic growth factors/cytokines that are necessary for neo-angiogenesis and repairing vascular damage in CLI and other ischaemic indications. Because the pooled population of human BMMSCs has been shown to increase blood flow in human clinical trials and in the pre-clinical animal models, we wanted to determine the angiogenic expression and secretion profile of our phBMMSCs. The screening of pro-angiogenic genes such as VEGF, HGF, Ang-1, SDF-1/CXCL12, IL-6, IL-8 and TGF-β1 by quantitative real time polymerase chain reaction (qRT-PCR) showed that all six batches of phBMMSCs expressed higher levels of mRNAs corresponding to VEGF (1.19 ± 0.17), HGF (409 ± 187), Ang-1 (100 ± 34.0), IL-6 (3.0 ± 1.2), IL-8 (39.0 ± 19.0) and TGF-β1 (0.59 ± 0.49) compared with the HFF cell line, which was used as control. The only exception was that the HFF cells expressed higher levels of SDF-1 mRNA than phBMMSCs (–0.44 ± 0.02). We also observed less variation in VEGF expression among the phBMMSC batches tested (Fig. 1a). The CM from the phBMMSCs (*n* = 6) was further evaluated for secretome profile using the growth factor array kit. We observed predominant expression of VEGF and insulin growth factor binding protein 6 (IGFBP-6) with an arbitrary unit of 20,855 ± 3566 and 22,522 ± 3528, respectively (Fig. 1b). In addition to the array analysis, we also quantified the selected angiogenic cytokines secreted by the same six batches of phBMMSCs in which the angiogenic gene expression was quantified. The cells secreted high levels of VEGF (2.6 ± 0.7 ng/ml/million cells; %CV = 25.2), HGF (31.8 ± 40 pg/ml/million cells; %CV = 78.2), SDF-1α (0.7 ± 0.54 ng/ml/million cells; %CV = 73.6), Ang-1 (1.3 ± 1.15 ng/ml/million cells; %CV = 96.6), IL-6 (6.3 ± 3.6 ng/ml/million cells; %CV = 52.4), IL-8 (18.1 ± 14.2 ng/ml/million cells; %CV = 72.2) and TGF-β1 (1.2 ± 0.3 ng/ml/million cells; %CV = 28.6) (Fig. 1c). Upon discerning the consistency in the mRNA expression and the secretion level of VEGF, we further evaluated the VEGF secretion across several passages (P4–P7) (Fig. 1d). The secreted amounts of VEGF in the CM of phBMMSCs across these four passages were observed to be similar with no significant differences (Fig. 1d). Thus, the consistency we observed with regards to VEGF expression in the cells and secretion in the corresponding CM, in comparison with other angiogenic factors tested, led us to select VEGF as a surrogate potency marker and to further evaluate its role in different functional angiogenic assays.

### Validation of VEGF as a surrogate angiogenic potency marker for phBMMSCs

**Fig. 2:**
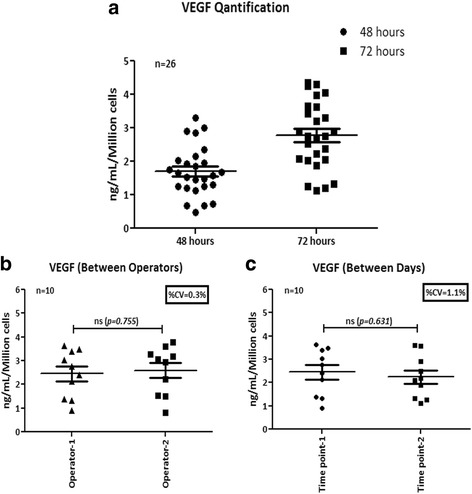
Quantification and validation of VEGF secreted by phBMMSCs. **a** VEGF levels in the CM from twenty six batches of phBMMSCs by ELISA. VEGF level at 48 h was 1.7 ± 0.7 ng/ml/million cells and at 72 h was 2.8 ± 1.0 ng/ml/million cells. **b** Quantification of VEGF levels in the CM from ten batches of phBMMSCs estimated by two different operators; no significant differences were observed in the VEGF levels estimated by two different operators determined by Student’s *t* test, *p* = 0.774. **c** Quantification of VEGF levels in the CM from ten batches of phBMMSCs estimated by a single operator on two different days; no significant differences were observed in the VEGF levels estimated by a single operator at different time points as determined by Student’s *t* test, *p* = 0.631. Data presented as mean ± SEM. SEMs shown as *error bars. VEGF* vascular endothelial growth factor

To determine the range of VEGF secretion by phBMMSCs in a large number of batches, we quantified the amount of VEGF in the CM from twenty six manufactured batches produced over a period of five years and several of these batches were used for pre-clinical studies and phase I and II clinical trials. The mean value of VEGF at 48 h was found to be 1.7 ± 0.7 ng/ml and that at 72 h was 2.8 ± 1.0 ng/ml for 1 × 10^6^ cells plated (Fig. 2a). We also determined the robustness of the assay method by quantifying VEGF in ten batches of phBMMSCs by two different operators on the same day and by a single operator performing the assay at two different time points for the same batches of CM (Fig. 2b, c). The data showed no significant differences between the two permutations and confirmed the reproducibility and consistency of the VEGF quantification assay.

### VEGF secreted by phBMMSCs induces signal transduction via the upregulation of VEGF receptors

**Fig. 3:**
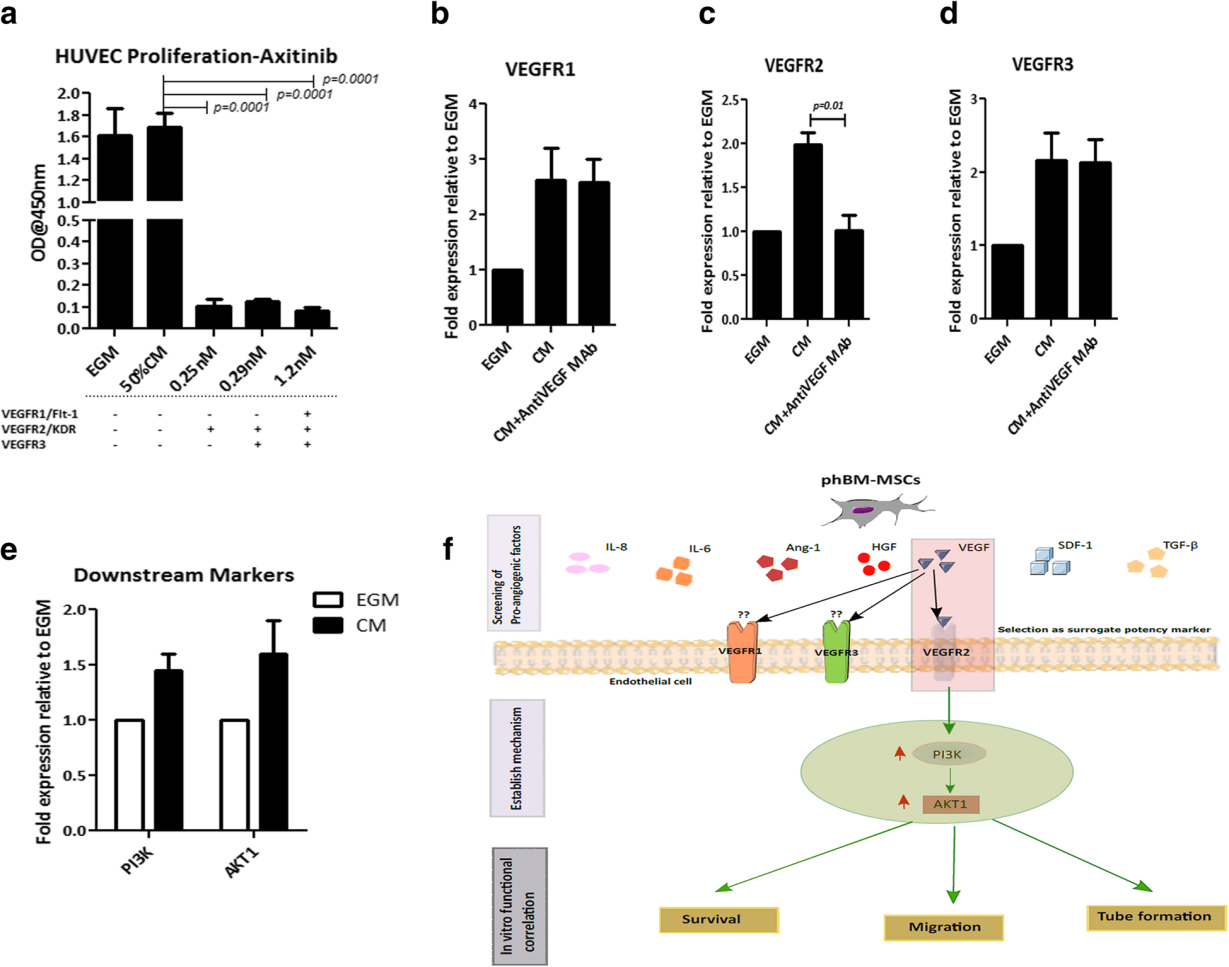
Activity of VEGF secreted from phBMMSCs is mediated by VEGFR2. **a** Proliferation of HUVECs using 50% CM from phBMMSCs and 50% CM with Axitinib at concentrations of 0.25 nM, 0.29 nM and 1.2 nM to block VEGFR2, VEGFR2 and VEGFR3, and VEGFR2, VEGFR3 and VEGFR1 respectively. Significant reduction in HUVEC proliferation was observed in the CM with all Axitinib treatment groups, ***p = 0.0001. EGM was used as a positive control for HUVEC proliferation. Analysis of VEGFR1 (**b**), VEGFR2 (**c**) and VEGFR3 (**d**) fold expression in HUVECs treated with phBMMSC CM and phBMMSC CM with anti-VEGF mAb by qRT-PCR. Significant reduction only in VEGFR2 expression was observed in the phBMMSC CM with anti-VEGF mAb group compared with the CM alone group, *p = 0.01. **e** Analysis of VEGF pathway-dependent markers PI3K and AKT1 in HUVECs cultured in the presence of phBMMSC CM by qRT-PCR; mRNA expression levels were normalized with housekeeping gene β-Actin as an internal standard and subsequently normalized to expression levels in HUVECs cultured in EGM as control. Data generated using two batches of phBMMSC CM, assayed in triplicates and presented as mean ± SEM. **f** Schematic diagram to depict the possible signal transduction pathway of VEGF secreted by phBMMSCs in HUVECs*.* SEMs shown as *error bars. HUVEC* Human umbilical vein endothelial cell, *EGM* endothelial growth medium, *CM* conditioned medium, *VEGFR* vascular endothelial growth factor receptor, *mAb* monoclonal antibody, *Ang-1* angiopoietin-1, *SDF-1* stromal-derived factor-1, *HGF* hepatocyte growth factor, *IL* interleukin, *TGF-β* transforming growth factor beta

Based on the accumulated literature evidence on the role of VEGF in inducing angiogenesis, we wanted to determine the mechanism by which such functions are brought about by phBMMSCs. To elucidate the VEGF receptor-mediated signalling, we blocked the VEGF receptors in the HUVECs using Axitinib [25], a small molecule that is known to inhibit VEGF receptors 2, 3 and 1 at concentrations of 0.25 nM, 0.29 nM and 1.2 nM, respectively. Upon blocking the VEGF receptors, the cells were allowed to proliferate in the presence of the CM obtained from phBMMSCs: we observed 102 ± 7.7% proliferation in the 50% CM group, considering the EGM group as 100% proliferation. HUVEC proliferation in the 50% CM with Axitinib at concentrations of 0.25 nM (VEGFR2), 0.29 nM (VEGFR2, VEGFR3) and 1.2 nM (VEGFR2, VEGFR3, VEGFR1) were 6.4 ± 1.8%, 7.6 ± 0.7% and 4.9 ± 1.1% respectively; these differences were not found to be statistically significant although the highest concentration of Axitinib showed maximum inhibition of HUVEC proliferation (Fig. 3a). Because we could not determine which receptor was specifically involved in the process, we further evaluated the mRNA expression of all three VEGF receptors on the HUVECs primed with the same CM. We observed a considerable upregulation of all three VEGF receptors. However, neutralization of secreted VEGF using anti-human VEGF mAb did not abolish the expression of VEGFR1 and VEGFR3, whereas a significant downregulation of VEGFR2/KDR was evident (*p* = 0.01) (Fig. 3b–d) Furthermore, an upregulation of the downstream genes, PI3K and AKT-1, was observed (Fig. 3e), thus confirming that the mechanism of VEGFR2-mediated signalling is operative for the phBMMSC population in the regulation of angiogenesis. A schematic diagram to illustrate this phenomenon is shown (Fig. 3f).

### Confirmation of the functional role of VEGF through in vitro angiogenic assays

**Fig. 4:**
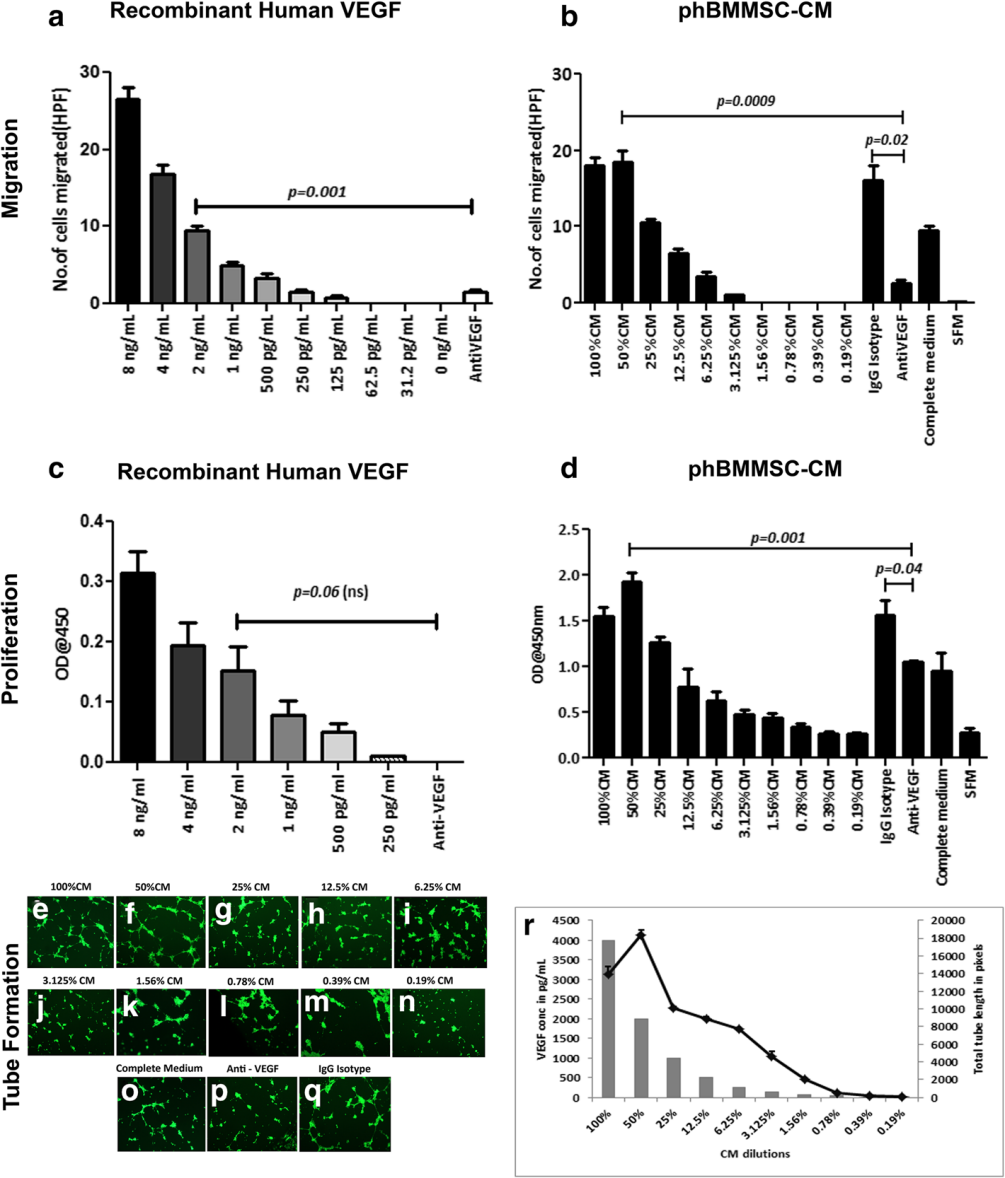
Functional in vitro HUVEC migration, proliferation and tube formation with rhVEGF and phBMMSC CM, followed by inhibition of VEGF activity. **a** Transwell migration of HUVECs in response to rhVEGF serially diluted from 8 ng/ml to 31.2 pg/ml in SFM. Significant inhibition of HUVEC migration was observed in 2 ng/ml rhVEGF with anti-VEGF mAb (10 μg/ml) compared to migration observed with 2 ng/ml rhVEGF concentration, **p = 0.001. **b** Transwell migration of HUVECs in response to phBMMSC CM serially diluted with SFM from 100% (4 ng/ml of secreted VEGF) to 0.19% (7.8 pg/ml of secreted VEGF). The maximum migration of HUVECs was observed at 50% dilution comprising 2 ng/ml secreted VEGF which was significantly inhibited by anti-VEGF mAb (10 μg/ml) in comparison with phBMMSC CM + IgG isotype, *p = 0.02, and phBMMSC CM, **p = 0.0009. Threshold for HUVEC proliferation was observed at 3.125% CM dilution with 125 pg/ml VEGF. **c** Proliferation of HUVECs in response to rhVEGF serially diluted starting from 8 ng/ml in SFM. rhVEGF did not effectively induce proliferation and therefore no measurable effect was observed with the anti-VEGF mAb. **d** HUVEC proliferation with phBMMSC CM serially diluted from 100% to 0.19% in SFM. Addition of anti-VEGF mAb to the 50% CM significantly reduced proliferation, **p = 0.001, in comparison with CM containing IgG isotype, *p = 0.04. Proliferation in HUVECs initiated at 0.78% CM dilution with 31.2 pg/ml VEGF. **e**–**q** HUVEC tube formation in response to phBMMSC CM serially diluted from 100% to 0.19%. Maximum tube formation was observed at 50% dilution containing 2 ng/ml secreted VEGF. Significant reduction in tube formation was observed when anti-VEGF mAb was used in 50% diluted CM, while IgG isotype did not alter the tube formation of HUVECs. **r** Graphical representation of tube formation induced by phBMMSC CM at various concentrations. Tube formation seemed to initiate at 0.78% CM dilution. Statistical significance was determined using Student’s *t* test. Data presented as mean ± SEM. SEMs shown as *error bars. CM* conditioned medium, *phBMMSC* pooled human bone marrow derived mesenchymal stromal cell, *SFM* serum-free medium, *VEGF* vascular endothelial growth factor, *HPF* high-power field

To further confirm that the VEGF secreted by phBMMSCs functionally induces angiogenesis, we performed parallel experiments using rhVEGF and the CM from the phBMMSC population. For this purpose, several HUVEC primary lines were generated and characterized for the expression of endothelial cell specific markers CD31, CD34 and vWF by flow cytometry and RT-PCR (Additional file 2: Figure S2). HUVECs harvested at P3–P5 were used for all in vitro assays. We performed HUVEC Transwell migration assays using rhVEGF solution in SFM at dilutions ranging from 8 ng/ml to 31.2 pg/ml (Fig. 4a). Except for the 8 ng/ml concentration, all other rhVEGF dilutions matched the concentration of secreted VEGF in the phBMMSC CM serially diluted from 100% (4 ng/ml VEGF) to 0.19% (7.8 pg/ml) (Fig. 4b). The 50% diluted phBMMSC CM, which contained 2 ng/ml of secreted VEGF brought about 51% greater migration than the 2 ng/ml rhVEGF (Fig. 4a, b). As expected, a dose-dependent reduction in the migrated cells was observed in both the rhVEGF and secreted VEGF groups, while the threshold concentration of VEGF necessary for HUVEC migration was determined to be 125 pg/ml. The neutralization of rhVEGF and the secreted VEGF using an anti-VEGF-blocking antibody at a concentration of 10 μg/ml inhibited HUVEC migration by 84% in the 2 ng/ml group of rhVEGF and by 85% inhibition in the 2 ng/ml secreted VEGF compared with the respective IgG isotype values. No significant difference in the migration of HUVECs was observed between the CM-treated and the isotype-treated cells (Fig. 4b).

Next, we compared the ability of rhVEGF and VEGF secreted by phBMMSCs with matched concentrations to determine the effect of VEGF in HUVEC proliferation. The rhVEGF alone in the SFM promoted the proliferation of HUVECs (Fig. 4c); however, considerably higher proliferation was observed when the HUVECs were incubated in the presence of CM (Fig. 4d). The CM from the phBMMSCs demonstrated a dose-dependent reduction in proliferation ranging from a 50% CM containing 2 ng/ml secreted VEGF to a 0.78% CM with 31.2 pg/ml of secreted VEGF. Neutralization of the secreted VEGF using the anti-VEGF mAb at a concentration of 10 μg/ml inhibited HUVEC proliferation up to 31% as compared with CM incubated with IgG isotype, thus indicating that HUVEC proliferation was partially mediated by VEGF (Fig. 4d). Similar to the results obtained with migration, we did not observe any difference in proliferation of HUVECs when incubated with CM and CM with IgG isotype.

We further investigated the role of VEGF in the final stage of in vitro angiogenesis by performing the tube formation assay in GFR Matrigel. We observed that rhVEGF alone at concentrations ranging from 8 ng/ml to 15.6 pg/ml did not promote the tube formation of HUVECs in GFR Matrigel (data not shown). Conversely, the CM from phBMMSCs significantly promoted tube formation in a dose-dependent manner, ranging from 50% (2 ng/ml of VEGF) to the lowest concentration of 0.78% (31.2 pg/ml VEGF) where initiation of tube formation was detected (Fig. 4e–q). It should be mentioned that 2 ng/ml of VEGF containing CM showed maximum tube formation (Fig. 4r). Addition of the anti-VEGF mAb (10 μg/ml) (Fig. 4p) inhibited tube formation by >50% compared with the CM group incubated with IgG isotype. Similar to the results obtained with migration and proliferation of HUVECs, tube formation was also unaffected with the addition of the IgG isotype. Taken together, these functional studies demonstrate clearly that the VEGF present in the CM was able to elicit migration, proliferation and tube formation of HUVECs in vitro (Fig. 4).

### Validation of VEGF-mediated in vitro angiogenic function by phBMMSC batches

**Fig. 5:**
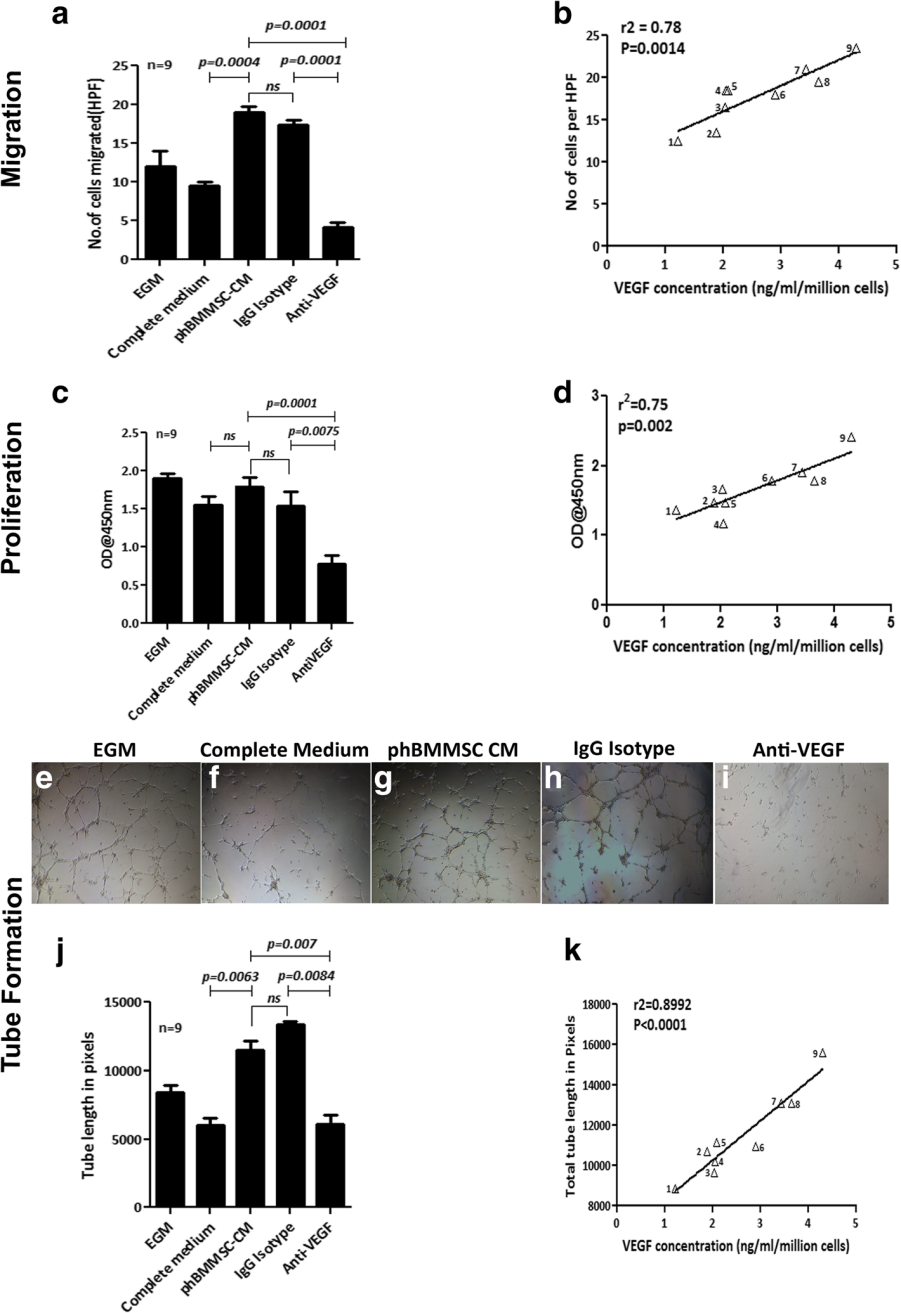
In vitro pro-angiogenic function of secreted VEGF from nine batches of phBMMSCs. **a** Evaluation of HUVEC migration under various culture conditions. CM (*n* = 9) induced significant migration of HUVECs. Migration was unaffected by an isotype-matched antibody (*p* = 0.1964), while addition of anti-VEGF mAb caused significant (*p = 0.0001) inhibition of cell migration. Migration of HUVECs in the CM group was significantly higher than that of control medium, ***p = 0.0004. **b** Correlation of VEGF concentration in phBMMSC CM (*n* = 9) with HUVEC migration was observed, *r* ^2^ = 0.78, **p = 0.0014. **c** HUVEC proliferation in response to various culture conditions; phBMMSC CM (*n* = 9) induced HUVEC proliferation similar to that of EGM and complete medium, although significant reduction of cell proliferation was observed in the CM group with anti-VEGF mAb in comparison to CM with isotype IgG, **p = 0.0075. Addition of IgG isotype in the CM did not alter HUVEC proliferation compared with CM alone, *p* = 0.2985. **d** Correlation of VEGF concentration-dependent proliferation of HUVECs was observed with phBMMSC CM (*n* = 9) with a high correlation coefficient, *r* ^2^ = 0.75, **p = 0.002. Representative images of tube formations in **e** EGM, **f** control medium, **g** phBMMSC CM, **h** IgG isotype, **i** phBMMSC CM with anti-VEGF mAb, 4× objective were used for magnification. **j** phBMMSC CM (*n* = 9) induces significant tube formation in HUVECs compared with EGM and control medium (**p = 0.0063). Tube formation was greatly reduced with the addition of anti-VEGF mAb in the CM in comparison with the isotype-matched control group (**p = 0.0084). Tube formation in HUVECs was unaffected with the addition of IgG isotype (*p* = 0.2985). **k** Concentration of VEGF in nine batches of CM highly correlated to the tube lengths, *r* ^2^ = 0.89, ***p = 0.0001. Data presented as mean ± SEM. SEMs shown as error bars*. CM* conditioned medium, *phBMMSC* pooled human bone marrow derived mesenchymal stromal cell, *EGM* endothelial growth medium, *VEGF* vascular endothelial growth factor, *HPF* high-power field﻿

To confirm that VEGF is indeed the angiogenic surrogate marker for the phBMMSC population, we evaluated the potential of the secreted VEGF in the CM derived from nine phBMMSC batches (Fig. 5). The biological assays performed were endothelial cell migration, proliferation and tube formation, which represent the entire process of angiogenesis and correlated with the functional activity through the secreted VEGF from each batch of phBMMSCs. First, HUVEC migration was assessed using the CM from all nine batches of phBMMSCs and the results are shown in Fig. 5a, b. We observed that the cell migration was comparable amongst all batches with a 19% coefficient of variation (Fig. 5b). Upon neutralization of VEGF, the migration of HUVECs was inhibited by >75% compared with the corresponding IgG isotype (Fig. 5a). The correlation of VEGF concentration in each of the nine batches with the number of migrated cells displayed high linearity with a correlation coefficient of 0.78 (*p* = 0.0014) (Fig. 5b).

Similarly, we also assessed the proliferation of HUVECs using all nine batches of CMs. The proliferation of HUVECs was comparable for all batches with a coefficient of variation of 21% (Fig. 5c). Neutralization of VEGF in the CM from all of the batches significantly blocked proliferation of HUVECs by >50% compared with the corresponding IgG isotype. The correlation of VEGF concentration quantified from these batches highly correlated with HUVEC proliferation and was calculated to generate a correlation coefficient value of 0.75 (*p* = 0.002) (Fig. 5d).

Finally, the in vitro angiogenic tube formation by HUVECs was assessed for these nine CM batches and the results are shown in Fig. 5. The extent of tube formation in all of the tested conditions are shown in Fig. 5e–i. It is clear from the representative images that the maximum tube length was observed in phBMMSC CM (Fig. 5g), phBMMSC CM with IgG isotype (Fig. 5h) and EGM (Fig. 5e) conditions, while complete medium and CM with anti-VEGF mAb showed similar extent of vessel formation (Fig. 5f and i respectively). Furthermore, a reduction of tube formation by >50% was observed in the presence of anti-VEGF mAb, while no such inhibition was observed with the IgG isotype (Fig. 5j). The average of total tube lengths obtained from all nine batches of phBMMSC CM and other conditions are graphically represented in Fig. 5j. Similar to migration and proliferation, a strong correlation (*r* ^2^ = 0.8992, *p* = 0.0001) was observed between the VEGF concentration and the tube lengths (Fig. 5k) with a low CV of 18%. Collectively, the data generated by three different in vitro functional measurements of angiogenesis correlated linearly with the concentration of VEGF present in the CM generated from the phBMMSC population.

## Discussion

Ischaemia is described as the shortage of oxygen in tissues or organs because of the restriction of blood supply instigated by vasoconstriction, thrombosis, embolism or vessel rupture in indications such as peripheral arterial disease and its more severe form, CLI [26]. In CLI patients there is extensive tissue damage due to severe blockage or degeneration of blood vessels and these patients often require therapeutic intervention using pro-angiogenic therapy. Moreover, CLI is associated with non-healing ulcers, gangrene and limb amputations which lead to increased morbidity and mortality rates [27]. Because conventional therapy is of limited value to these patients, attempts are being made to determine whether cell and gene therapy can provide sustainable benefit to CLI patients.

Cell-based therapeutic angiogenesis is gaining momentum as an alternative to the current revascularization strategies. Several pre-clinical and clinical studies using EPCs, autologous CD34^+^ BMMNCs and autologous or allogeneic MSCs derived from various tissue sources including bone marrow have demonstrated their interaction with endogenous endothelial cells via paracrine cross-talks or direct cell-to-cell interaction, thus promoting angiogenesis, resulting in limb salvage [6–8, 28]. The first clinical trial for therapeutic angiogenesis using cell transplantation (TACT) in PAD patients with autologous BMMNCs and peripheral blood mononuclear cells, conducted successfully by Tateishi-Yuyama et al. in 2002 [8], showed evidence of improvement in multiple clinical parameters in the patients injected with BMMNCs. Since then, a number of clinical trials using a variety of cell types have been completed with different degrees of success [29–32]. A trial using autologous BMMNCs showed greater clinical improvements in patients with Buerger’s disease in comparison with those with atherosclerotic PAD, and this was associated with greater EPC migration in response to VEGF in Buerger’s disease patients [33]. In our own phase I/II clinical trial where we have established the safety of phBMMSC administration in CLI patients, we also observed that there is a significant improvement in the ankle brachial pressure index (ABPI) in the cell-treated group of patients compared with placebo [19]. Recently, we have published our phase II clinical data demonstrating that intra-muscular administration of phBMMSCs in Buerger’s disease CLI patients resulted in a significant decrease in rest pain and improvements in ulcer healing and blood circulation [20]. Moreover, new collateral vessel formation was also observed in some of these patients suggesting that the cells were able to initiate neo-angiogenesis. As we progress towards a phase III clinical trial with CLI patients, in addition to developing identity, purity and safety assays, it is critical to develop an angiogenesis-specific potency assay, intended to be applied for disease indications that require therapeutic angiogenesis, to demonstrate that the expression of the ‘so-called’ potency marker by the cell product corroborates with the therapeutic outcome. Potency assay development requires establishing a clear connection between the therapeutic cells and their functional activity specific to the intended clinical indication [34]. It is necessary to develop such an assay or assay matrix for allogeneic MSCs in a progressive manner from the pre-clinical stage through various clinical trial phases and implemented at the time of phase III trial.

The United States Food and Drug Administration (US FDA) and European Medicines Agency (EMA) accept non-functional assays for potency testing, but it is essential to develop an in vitro or in vivo biologically functional assay and attempt to correlate the potency of the product, at least to the in vitro functional assay. Correlating the potency results with the pre-clinical or clinical outcomes is usually challenging because of their complex, multimodal mechanism of action, particularly where live cells are used for treatment.

In this study, based on the existing literature supporting the therapeutic benefit of BMMSCs in revascularization, the expression of pro-angiogenic genes was initially screened in our pooled cell population and the results clearly demonstrated their pro-angiogenic predisposition (Fig. 1). The clear demonstration of the presence of pro-angiogenic growth factors and cytokines at the gene and protein levels suggested that phBMMSCs can be a therapeutically beneficial product for treating diseases in which tissue angiogenesis is severely impaired. Among the various factors we have screened and estimated, VEGF appears to be expressed consistently and secreted by these cells in the CM. In addition to VEGF, phBMMSCs secrete varying amounts of other well characterized angiogenic growth factors like Ang-1, SDF-1, HGF, IL-6, IL-8 and TGF-β1. Many of these factors have been shown to have a specific role in the entire cascade of ‘physiological angiogenesis’, without which adequate vascularization may not be possible [35]. The comparable levels of VEGF secretion by the phBMMSCs across various passages suggested that the regulation of VEGF synthesis is well controlled in the manufactured phBMMSC product (Fig. 2). These observations led us to choose VEGF as a surrogate potency marker for phBMMSCs that has long been identified as the primary regulator of angiogenesis [36, 37]. It is noteworthy that the significance of VEGF in inducing pro-angiogenesis has not only been restricted to MSCs, because human neural stem cells (hNSCs) have also been reported to support angiogenesis in vitro and in vivo and the proposed mechanism of action is probably governed by VEGF [38]. Quantitation of VEGF on twenty six manufactured batches over a period of five years suggested stability as well as reproducibility of VEGF secretion by the manufactured and cryopreserved phBMMSCs; similar data pertaining to VEGF were shown earlier with Multistem®, a large-scale expanded multipotent adult progenitor cell (MAPC) product [39]. Based on the VEGF quantitation data obtained from many production batches (Fig. 2), the level of VEGF may be estimated to be ≥2 ng/ml/million phBMMSCs to qualify these cells for eliciting neo-angiogenic activity.

VEGF is known to bind to its receptors, VEGFR1/Flt-1, VEGFR2/KDR and VEGFR3, with differential affinity and associated functions on EPCs [40, 41]. In order to elucidate the specificity of the receptor binding of the VEGF secreted by phBMMSCs, we used Axitinib, a specific receptor tyrosine kinase blocker, at different concentrations to block the three VEGF receptors [25]. Our results clearly suggest that VEGF synthesized by phBMMSCs specifically binds to VEGFR2, because >90% of VEGF binding was abolished in the presence of Axitinib at the lowest concentration of 0.25 nM, which is precisely preordained to neutralize VEGFR2 activation. Additionally, the expression of all three VEGF receptors was upregulated in the endothelial cells upon exposure to the CM from phBMMSCs, while neutralization of the secreted VEGF in the CM significantly downregulated only VEGFR2 expression and not the other two receptors, thus indicating the specificity of VEGF secreted by the phBMMSCs to VEGFR2. Although VEGFR2 has a lower binding affinity in comparison with VEGFR1, VEGFR2 has nevertheless been reported to be the main transducer for VEGF-A driving endothelial cell survival, migration, proliferation and their differentiation during vascular tube formation [41–43]. The survival of endothelial cells in ischaemic tissues is a key requirement in therapeutic angiogenesis, and thus we looked into the survival-related downstream molecules PI3K and AKT. In our results, we observed an increased expression of PI3K and AKT in the CM-primed endothelial cells, which further indicates the specificity of VEGF in promoting endothelial cell survival.

The contribution of VEGF was also evaluated using rhVEGF alongside the CM generated by culturing phBMMSCs at various concentrations to understand the implication of VEGF in the angiogenic process. VEGF contributed majorly by facilitating in vitro migration, proliferation and tube formation of HUVECs (Figs 4 and 5). Biological assays along with the non-biological potency assay (e.g. ELISA) could add value to the potency evaluation of the therapeutic product, and these assays could be part of the potency assay matrix. It is also important to note that Stempeucel® has been shown to ameliorate foot necrosis and limb salvage in an in vivo murine model of hind-limb ischemia [20, 44]. Our next logical approach is to determine whether blocking VEGF immediately after Stempeucel® administration would obviate neo-angiogenesis in a felicitous pre-clinical model.

The data presented here suggest a high correlation of VEGF concentration with in vitro endothelial function, indicating that prior estimation of VEGF by ELISA can be considered a categorical, reliable and robust surrogate marker for angiogenic potency, quantifiable outside a living system, and also satisfies all regulatory requisites for a large-scale, expanded, allogeneic BMMSC population, Stempeucel®.

## Conclusion

In this article, we have followed a systematic approach towards developing and qualifying a potency assay as per the regulatory requirements by screening, estimating and selecting a single surrogate marker for angiogenesis. Consistency of the assay was also substantiated by establishing the reproducibility and mechanism of action as well as correlating with in vitro functional assays. Taken together, we propose that VEGF can be considered a single surrogate marker for angiogenic potency and set an example for developing and validating a potency assay for clinical-grade allogeneic phBMMSCs intended for therapeutic angiogenesis in CLI and other vascular diseases.

## Additional files

Additional file 1: Figure S1. showing **a** characterization of phBMMSCs for MSC-positive surface markers (CD44, CD73, CD90, CD105 and CD166) and negative markers (CD14, CD19, CD34, CD45, CD133 and HLA-DR), and indication of viability (*inset*), by flow cytometry; **b** differentiation of phBMMSCs into adipocytes, osteocytes and chondrocytes, stained with Oil Red O, Alizarin Red and Saffranin O, respectively. (TIF 6151 kb)
Additional file 2: Figure S2. showing **a** morphology of HUVECs at P1; **b–d** characterization of HUVECs using three-colour flow cytometry analysis for surface markers CD31, CD34 and CD73 indicating high HUVEC purity; **e** characterization of HUVECs by reverse transcriptase PCR for endothelial specific markers, vWF (von Willebrand factor), VE-Cadherin, CD34 and CD31. (TIF 3909 kb)
Additional file 3: Table S1a. presenting the layout of a chemiluminescence detection-based growth factor array for Fig. 1b; information provided by the manufacturer. (TIF 1976 kb)

## Abbreviations

ABPI: Acute brachial pressure index
AKT/PKB: Protein kinase B
Ang-1: Angiopoietin-1
bFGF: Basic fibroblast growth factor
BMMNC: Bone marrow mononuclear cell
CLI: Critical limb ischaemia
CTP: Cell therapy product
DMEM-KO: Dulbecco’s modified Eagle medium, knockout
EBM: Endothelial basal medium
EGM: Endothelial growth medium
ELISA: Enzyme-linked immunosorbent assay
EMA: European Medicines Agency
EPC: Endothelial progenitor cell
FBS: Fetal bovine serum
GFR: Growth factor reduced
HFF: Human foreskin fibroblast
HGF: Hepatocyte growth factor
HUVEC: Human umbilical vein endothelial cell
IGFBP-6: Insulin like growth factor binding protein-6
IL: Interleukin
mAb: Monoclonal antibody
MAPC: Multipotent adult progenitor cell
MNC: Mononuclear cell
MSC: Mesenchymal stromal cell
PAD: Peripheral arterial disease
PBMNC: Peripheral blood mononuclear cell
phBMMSC: Pooled human bone marrow-derived mesenchymal stromal cell
PI3K: Phosphoionositide-3, kinase
qRT-PCR: Quantitative reverse transcriptase polymerase chain reaction
rhVEGF: Recombinant human vascular endothelial growth factor
SDF-1: Stromal-derived factor-1
TACT: Therapeutic angiogenesis using cell transplantation
TGF-β1: Transforming growth factor beta-1
US FDA: United States Food and Drug Administration
VEGF: Vascular endothelial growth factor
VEGFR: Vascular endothelial growth factor receptor
vWF: Von Willebrand factor
