# Supplementary figures and images for: Development of a surrogate potency assay to determine the angiogenic activity of Stempeucel®, a pooled, ex-vivo expanded, allogeneic human bone marrow mesenchymal stromal cell product
Source: Stem Cell Res Ther. 2017 Feb 28;8:47. doi: 10.1186/s13287-017-0488-3 (PMC5331748; doi:10.1186/s13287-017-0488-3)

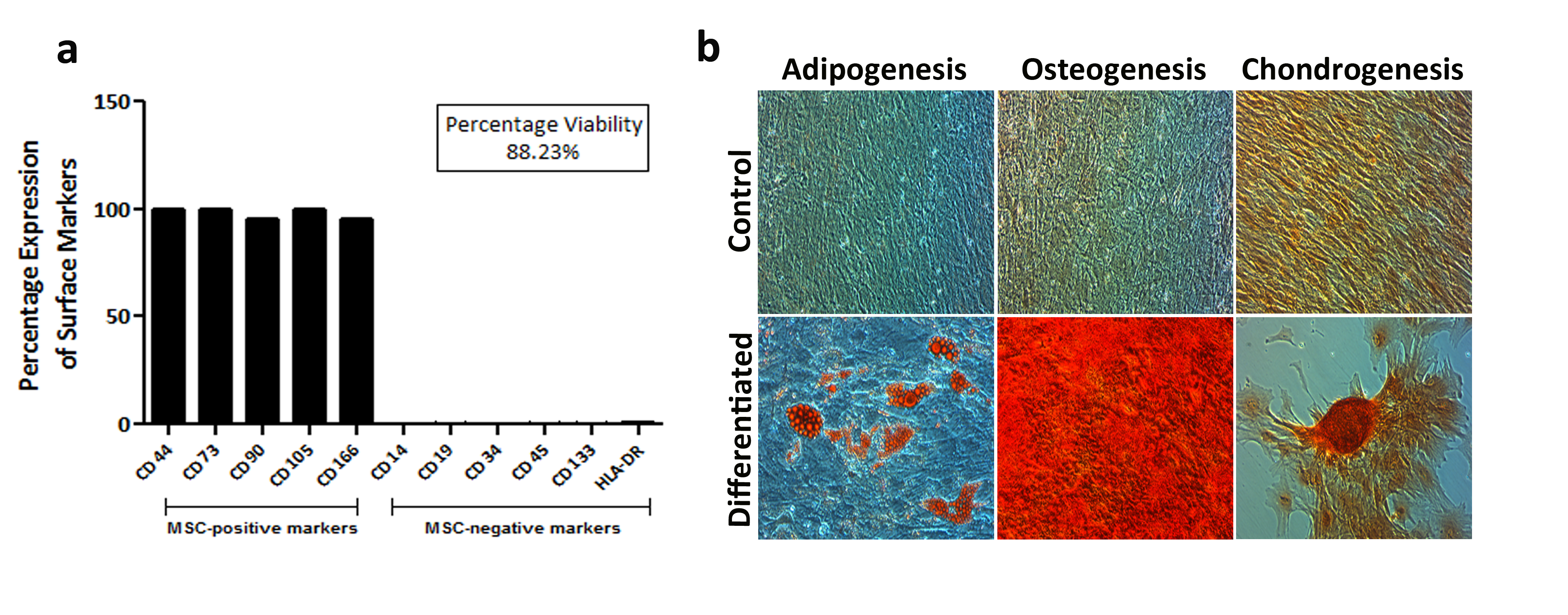

Supplement: Additional file 1: Figure S1. — showing a characterization of phBMMSCs for MSC-positive surface markers (CD44, CD73, CD90, CD105 and CD166) and negative markers (CD14, CD19, CD34, CD45, CD133 and HLA-DR), and indication of viability (inset), by flow cytometry; b differentiation of phBMMSCs into adipocytes, osteocytes and chondrocytes, stained with Oil Red O, Alizarin Red and Saffranin O, respectively. (TIF 6151 kb) [file 13287_2017_488_MOESM1_ESM.tif]

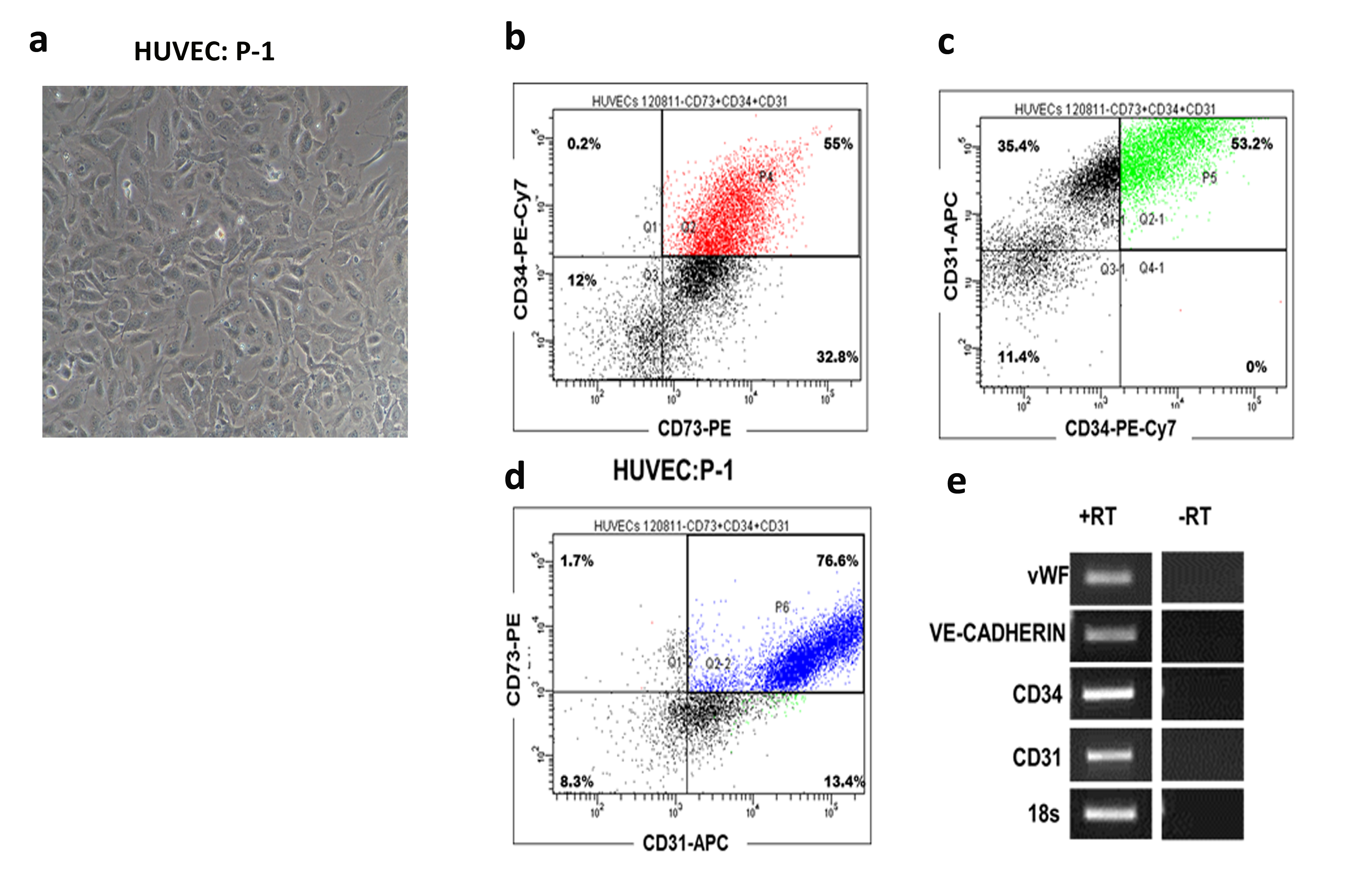

Supplement: Additional file 2: Figure S2. — showing a morphology of HUVECs at P1; b–d characterization of HUVECs using three-colour flow cytometry analysis for surface markers CD31, CD34 and CD73 indicating high HUVEC purity; e characterization of HUVECs by reverse transcriptase PCR for endothelial specific markers, vWF (von Willebrand factor), VE-Cadherin, CD34 and CD31. (TIF 3909 kb) [file 13287_2017_488_MOESM2_ESM.tif]

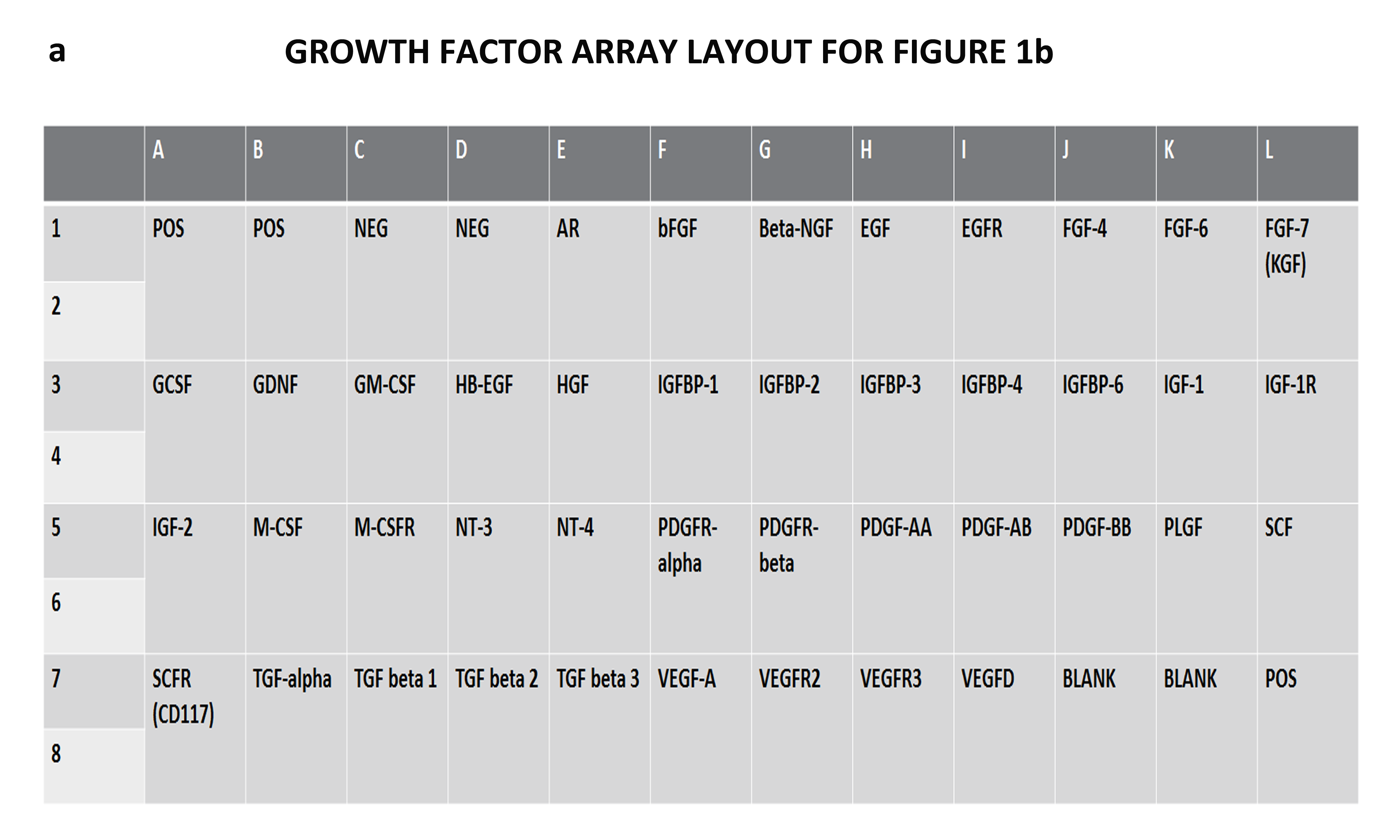

Supplement: Additional file 3: Table S1a. — presenting the layout of a chemiluminescence detection-based growth factor array for Fig. 1b; information provided by the manufacturer. (TIF 1976 kb) [file 13287_2017_488_MOESM3_ESM.tif]
